# A novel SMARCAL1 mutation associated with a mild phenotype of Schimke immuno-osseous dysplasia (SIOD)

**DOI:** 10.1186/1471-2369-15-41

**Published:** 2014-03-03

**Authors:** Luisa Santangelo, Maddalena Gigante, Giuseppe Stefano Netti, Sterpeta Diella, Flora Puteo, Vincenza Carbone, Giuseppe Grandaliano, Mario Giordano, Loreto Gesualdo

**Affiliations:** 1 Unit of Pediatric Nephrology, University Hospital “Policlinico Consorziale - Giovanni XXIII”, Bari, Italy; 2 Center for Renal Immunopathology, Molecular Diagnostics and Regenerative Medicine, Bari-Foggia, Italy; 3 Unit of Nephrology, Dialysis and Transplantation, Department of Emergency and Organ Transplantation - University “Aldo Moro”, Bari, Italy

**Keywords:** Focal segmental glomerulosclerosis, Schimke immuno-osseus dysplasia, SMARCAL1, Mutational analysis

## Abstract

**Background:**

Schimke immuno-osseous dysplasia (SIOD, OMIM #242900) is an autosomal-recessive pleiotropic disorder characterized by spondyloepiphyseal dysplasia, renal dysfunction and T-cell immunodeficiency. SIOD is caused by mutations in the gene *SMARCAL1*.

**Case presentation:**

We report the clinical and genetic diagnosis of a 5-years old girl with SIOD, referred to our Center because of nephrotic-range proteinuria occasionally detected during the follow-up for congenital hypothyroidism. Mutational analysis of *SMARCAL1* gene was performed by polymerase chain reaction (PCR) and bidirectional sequencing. Sequence analysis revealed that patient was compound heterozygous for two *SMARCAL1* mutations: a novel missense change (p.Arg247Pro) and a well-known nonsense mutation (p.Glu848*).

**Conclusion:**

This report provided the clinical and genetic description of a mild phenotype of Schimke immuno-osseous dysplasia associated with nephrotic proteinuria, decreasing after combined therapy with ACE inhibitors and sartans. Our experience highlighted the importance of detailed clinical evaluation, appropriate genetic counseling and molecular testing, to provide timely treatment and more accurate prognosis.

## Background

Schimke Immuno-Osseous Dysplasia [SIOD; OMIM #242900] is a rare autosomal recessive multisystem disorder, firstly described in 1971 [1,2]. Approximately 50 cases have been reported in the literature so far, without any apparent sex, ethnic or geographic predilection. The exact prevalence of the disease is unknown, in North America the incidence is estimated at 1:1,000,000 to 1:3,000,000 live births. Typical findings of SIOD are spondyloepiphyseal dysplasia with disproportionate growth failure, typical facial appearance, nephrotic syndrome with focal segmental glomerulosclerosis (FSGS) and progressive renal failure, recurrent lymphopenia, T-cell immunodeficiency, and pigment naevi [1-3]. Other features include hypothyroidism, episodic cerebral ischaemia and bone marrow failure [4]. The SIOD phenotype may range from a severe variant with *in utero* onset to a milder form with later onset [5,6]. SIOD is caused by mutations in the gene encoding HepA-related protein (HARP) also known as *SMARCAL1* [SWI/SNF-related, matrix associated, actin-dependent regulator of chromatin, subfamily a-like 1; Gene ID: 50485; NG_009771.1], a protein homologous to the sucrose non fermenting type 2 (SNF2) family of chromatin-remodeling proteins, required for transcriptional regulation, replication, repair, recombination, and covalent modification [7-11]. Biallelic putative loss of function mutations in SMARCAL1 gene are the only identified causes of SIOD, however approximately half of patients referred for molecular studies have no detectable mutations in the coding region of this gene, thus environmental, genetic, or epigenetic modifiers and the existence of endophenotypes of SIOD have been hypothesized [10].

Here, we report the clinical and genetic diagnosis of a 5-years old girl with SIOD, referred to our Center because of nephrotic-range proteinuria occasionally detected during the follow-up for congenital hypothyroidism.

## Case presentation

**Figure 1:**
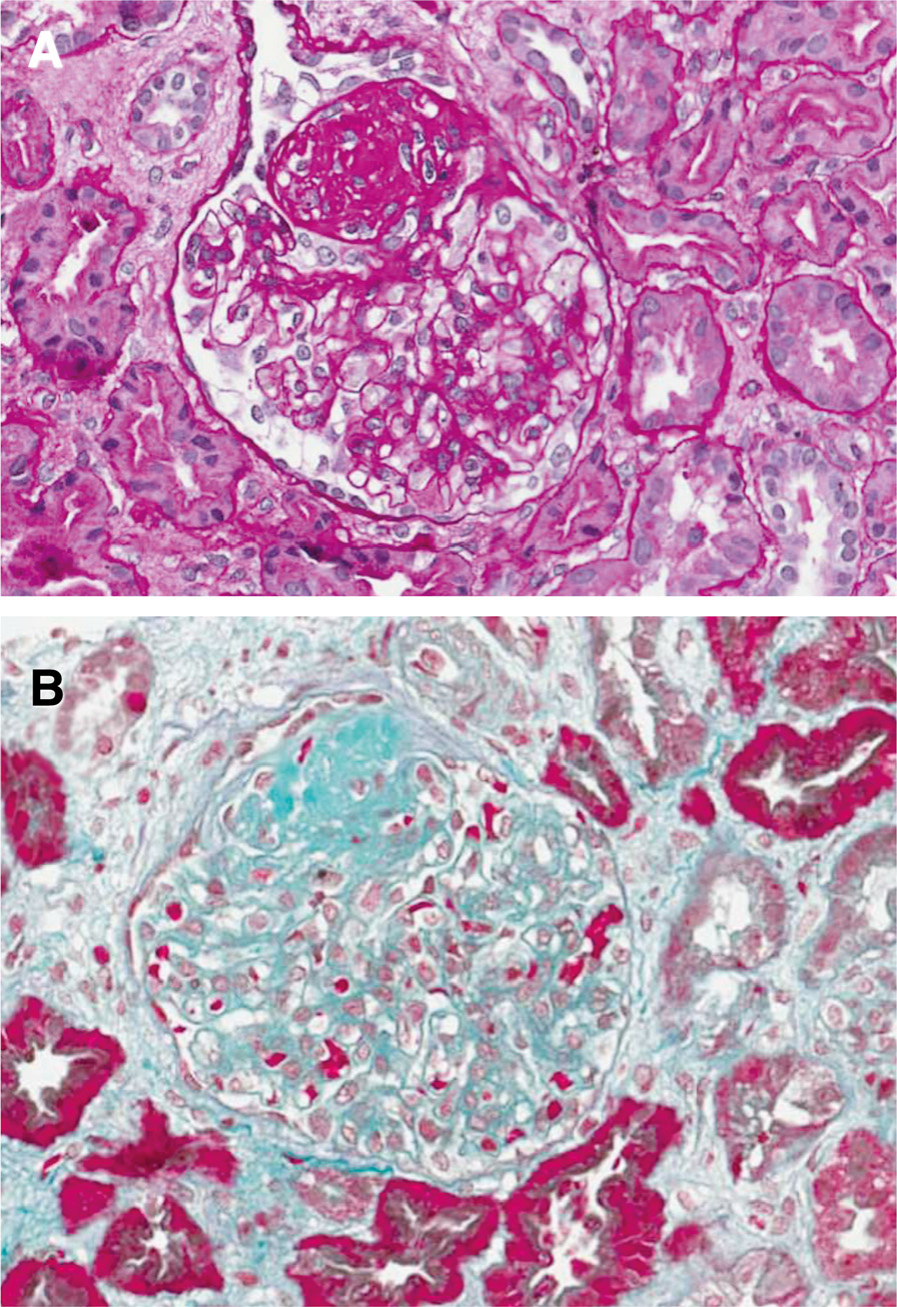
**Renal histology of SIOD patient.** Light microscopy images **(A, B)** showing larger glomerular volume, with a sclerotic lesion of the glomerular tuft. The remaining part of the glomerulus shows focal and segmental matrix increase and mesangial proliferation. Diffuse tubular atrophy and rare arteriolar ialinosis are also present (**A**: PAS staining, **B**: Masson’s Trichrome stain; ×400).

The patient was born at 29 week of gestation as the first child of healthy non-consanguineous Italian parents. Her birth weight was 720 g (<3^rd^ percentile). Congenital hypothyroidism was quickly diagnosed and substitutive therapy was started at birth time. At 5-years, she was referred to our Pediatric Nephrology Center. At clinical examination she had disproportionately short stature (94 cm; <3^rd^ percentile), low weight (13,5 kg; <3^rd^ percentile), reduced occipitofrontal head circumference (OFC) (48,1 cm; <3^rd^ percentile), dorsolumbar kyphoscoliosis, fine hair, pale skin, low nasal bridge. She had normal intelligence and never had severe infections, migraines or transient ischemic attacks. Moreover she did not present short neck or trunck, hyperpigmented macules, corneal opacities or hypertension. Laboratory data showed nephrotic range proteinuria (1,7 g/die; 125 mg/kg/die) and normal renal function (creatinine clearance 84,75 ml/min, according to the Schwartz-formula). Lymphopenia with T-cell deficiency was also detected. Skeletal radiograph revealed dorsolumbar kyphoscoliosis with unbalanced iliac crests, but other findings consistent with the diagnosis of spondyloepiphyseal dysplasia, such as ovoid and mildly flattened vertebral bodies, small deformed capital femoral epiphyses, and shallow dysplastic acetabular fossae, were absent. The renal biopsy was performed and revealed FSGS (Figure 1A-B). Treatment with ramipril and irbesartan resulted in a reduction of proteinuria (0,104 g/die). After two-years follow-up, the patient displays normal renal function without proteinuria and no episodes of infection or cerebrovascular complication.

On the basis of clinical and laboratory findings, a diagnosis of SIOD was suspected. After obtaining informed consent for genetic studies, genomic DNA was purified from peripheral blood samples of proband and all available family members (one sibling, mother and father) using standard procedures.

Mutational analysis of *SMARCAL1* gene [NM_014140.3 GI:187761312; NG_009771.1 GI:223671908; GeneID: 50485] was performed by polymerase chain reaction (PCR) and bidirectional sequencing of the coding exons and intron/exon flanking regions, as previously described [12]. *SMARCAL1* flanking intronic primers were designed using *primer3* program (http://primer3.wi.mit.edu/). PCR products were sequenced using the Big Dye Terminator v3.1 cycle sequencing kit on 3130 Genetic Analyzer (Life Technologies, Ltd). *SMARCAL1* mutation was named according to Human Genome Variation Society recommendations (http://www.hgvs.org/mutnomen) and NCBI Reference Sequence [NM_014140.3 GI:187761312]. The potential effect of novel missense mutation was analyzed using SIFT (Sorting Intolerant From Tolerant) programme [13] and Polyphen programme [14].

**Figure 2:**
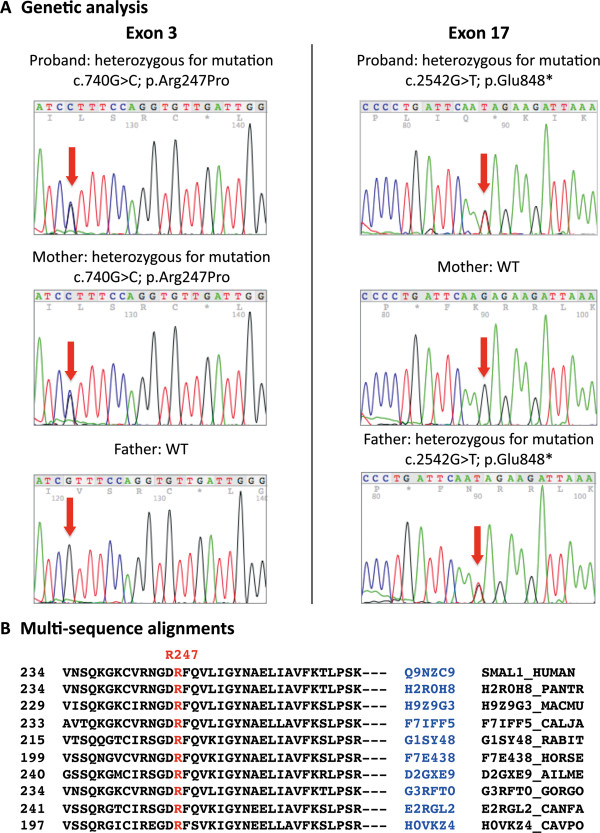
**Genetic analysis. A**. Electropherograms showing detected mutations of *SMARCAL1* gene [NM_014140.3 GI:187761312] in exons 3 and 17 of proband and parents. **B**. Multi-sequence alignments by ClustalW software.

Sequence analysis revealed that patient was compound heterozygous for two mutations (Figure 2A): a novel missense mutation in exon 3 (c.740G > C), resulting in an arginine-to-proline substitution (p.Arg247Pro), inherited by the mother; and a nonsense paternally-derived mutation in exon 17 (c.2542G > T) resulting in the substitution of Glu848 with a stop codon (p.Glu848*) [9]. The healthy brother was wild type for detected mutations (data not shown). This study have been performed in accordance with the Declaration of Helsinki and was approved by the Ethical Committee of University Hospital in Foggia.

## Conclusions

SIOD is a rare autosomal recessive pleiotropic disorder caused by mutations in *SMARCAL1* gene [1,2]. So far, about 55 different mutations in *SMARCAL1* gene have been identified in SIOD patients from different ethnic backgrounds [9,10,15,16]. The pathogenesis of SIOD is largely unknown. *SMARCAL1* gene encodes the HepA-related protein (HARP), a member of the SNF2 family of ATPases, acting as chromatin remodelers within multi-protein complexes [11]. This protein is an ATP-driven annealing helicase, involved in a wide range of biological functions, including transcription, DNA replication, and DNA repair. SIOD patients exhibit a continuum from mild to severe disease. Severe form is characterized by intrauterine growth retardation, severe growth failure after birth, recurrent infections, hematological abnormalities, hypothyroidism, cerebrovascular disease and often death within the first 15-years. Mild form usually displays growth failure and renal dysfunction between 8–12 years, without infections or cerebrovascular disease [4-6]. Of note, our patient fulfilled all the criteria for the mild phenotype of SIOD, i.e., absence of disease symptoms in the 1st year of life [1], growth failure and nephrotic syndrome starting in childhood [2], normal thyroid function tests and [5] no infectious or cerebrovascular symptoms until now [4,5]. However early recognition of characteristic growth retardation coupled with bone abnormalities may represent key clues for the diagnosis of this genetic disease even in mild form. Skeletal examination in these patients may show bone changes suggestive for spondyloepiphyseal dysplasia, driving the correct diagnosis.

Usually, patients with *SMARCAL1* biallelic missense mutations or a missense and a nonsense mutation have a milder disease. Accordingly, our patient, compound heterozygous for a novel missense mutation (p.Arg247Pro) and a well-known nonsense mutation (p.Glu848*) [9], displayed a mild form. The missense mutation (p.Arg247Pro) within the first HARP domain (HARP1), described for the first time in this report, is located in a highest-conserved site of the multi-sequence alignment (Figure 2B) and is predicted to be a damaging change by SIFT (Sorting Intolerant From Tolerant) programme [13] with a score of 0.0 and a ‘probably damaging’ substitution by Polyphen-programme [14] with a score of 1.000. All SNF2 proteins are characterized by the presence of SWI/SNF helicase motifs but do not always exhibit helicase activity. SMARCAL1 protein has ATP-dependent annealing helicase activity, which helps to stabilize stalled replication forks and facilitate DNA repair during replication. Recently, it was shown that the conserved tandem HARP (2HP) domain dictates this annealing helicase activity, suggesting that the HARP domains are important determinants of the SMARCAL1 enzyme specificity [17]. The nonsense mutation, p.Glu848*, leading to a truncated SMARCAL1 protein of 847 aa, was previously reported in other SIOD patients with different ethnic origin [9].

The patient was referred to our attention because of onset of proteinuria in nephrotic range. Renal biopsy revealed FSGS, which is the most frequent renal pathological finding associated with SIOD, as described in a revision of 39 SIOD cases with proteinuria [18]. Nevertheless cases of minimal change disease, membranous nephropathy, mesangial proliferative glomerulonephritis and nephrophthisis have been also described [15,18]. Kidney involvement in SIOD patients displays typically proteinuria evolving to overt nephrotic syndrome, usually diagnosed between 1–14 years [4-6,9]. This genetic form of nephrotic syndrome usually does not respond to steroid treatment [6,16]; nevertheless, transient reductions in proteinuria using ACE-inhibitors, NSAID or even cyclosporine-A have been documented [4-6,9]. Our experience demonstrate that nephrotic proteinuria associated with a mild form of SIOD may respond to combined therapy with ACE- inhibitors and sartans, supporting the concept that several missense mutations in *SMARCAL1* gene retain some residual function [15]. However most of patients, mainly with severe forms, progress to ESRD between 5–15 years of age. No relapse of proteinuria has been described in SIOD patients after renal transplantation [15], while the evolution of cerebrovascular and infectious complications do not seem to improve after transplantation.

In conclusion, we report a mild phenotypic expression of SIOD associated with a new genotype consisting of compound-heterozygosity for a known nonsense mutation and a novel *SMARCAL1* missense change, characterized by nephrotic proteinuria, which decreased after combined therapy with ACE inhibitors and sartans. Our experience highlighted the importance of detailed clinical evaluation, appropriate genetic counseling and molecular testing, to provide timely treatment and more accurate prognosis.

## Consent

Written informed consent was obtained from parents for publication of this case report. A copy of the written consent is available for review by the Editor-in-Chief of this journal.

## Abbreviations

HARP: HepA-related protein; PCR: Polymerase chain reaction; SIFT: Sorting intolerant from tolerant; SMARCAL1: SWI/SNF-related, matrix associated, actin-dependent regulator of chromatin, subfamily a-like 1; SNF2: Sucrose non fermenting type 2; NSAID: Nonsteroidal anti- inflammatory drugs; ACE-inhibitors: Angiotensin-converting-enzyme inhibitors.

## Competing interests

The authors declare that they have no competing interests.

## Authors’ contribution

LS, FP, VC and MG (MD) participated in clinical evaluation, MG (PhD) and SD carried out molecular genetic studies, GSN analyzed data and drafted the manuscript, GG helped to draft the manuscript, and LG participated in design and coordination of study and gave the final approval. All authors’ read and approved the final manuscript.

## Authors’ information

LS: post-graduate school in Nephrology, University of Foggia; MG (PhD): Post-graduate school in Medical Genetics, permanent position as Biologist, University of Foggia; GSN: MD PhD, Assistant Professor of Clinical Pathology, University of Foggia; SD: laboratory technician, University of Foggia; FP, VC and MG (MD): Staff physician, Pediatric Nephrology Unit, Ospedale “Giovanni XXIII”, Bari; GG: Associate Professor of Nephrology, University of Foggia; LG: Full Professor of Nephrology, University of Bari.

## Pre-publication history

The pre-publication history for this paper can be accessed here:

http://www.biomedcentral.com/1471-2369/15/41/prepub
